# Supplementary material for: Lineage plasticity enables low-ER luminal tumors to evolve and gain basal-like traits
Source: Breast Cancer Res. 2023 Mar 1;25:23. doi: 10.1186/s13058-023-01621-8 (PMC9979432; doi:10.1186/s13058-023-01621-8)
Supplement: Supplementary file 1 — Additional file 1. Supplementary figures 1–5. [file 13058_2023_1621_MOESM1_ESM.pdf]

Supplementary figures:

A

Histological features of 46 Low-ER positive carcinomas	
Type	Invasive ductal carcinoma: 96% (44)<br>Invasive lobular carcinoma: 4% (2)
Grade	High: 89% (41)<br>Intermediate: 11% (5)
Size	2.97 ± 2.6 cm (range: 0.1 – 10.0 cm)
Ductal carcinoma in-situ present	55%
Lymphovascular invasion present	39%
Lymph node status	N0 = 30 (ITCs in two patients)<br>N1 = 2 N2 = 0 N3 = 4
Response to neoadjuvant chemotherapy	Pathologic complete response = 75% (9/12)<br>Pathologic partial response = 25% (3/12)*<br>*Two had microinvasion only

Oncotype Recurrence Score (n=10) = 48.8 ± 12 (18-61)

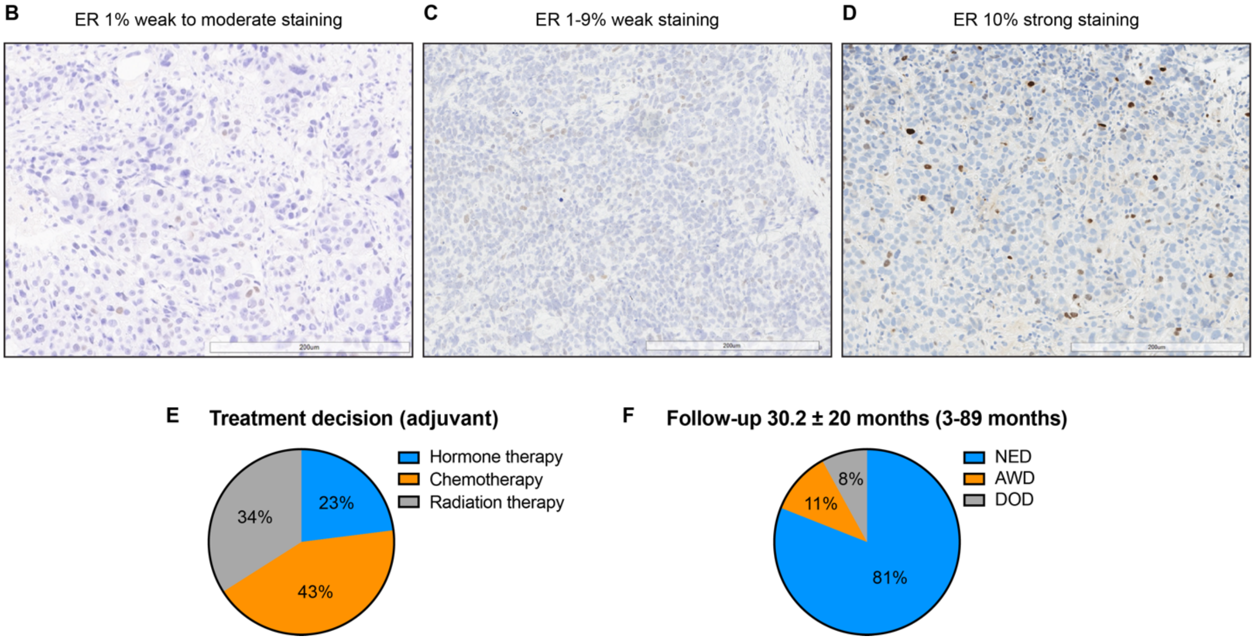

Supplementary Figure 1: (A) Histological features of the 46 low-ER cases in our cohort. **(B-D)** Representative IHC images showing the different levels and intensity of ER staining in the low-ER tumors. **(E-F)** Breakdown of treatment decisions **(E)**, and follow-up details **(F)**, of the 46 low-ER cases.

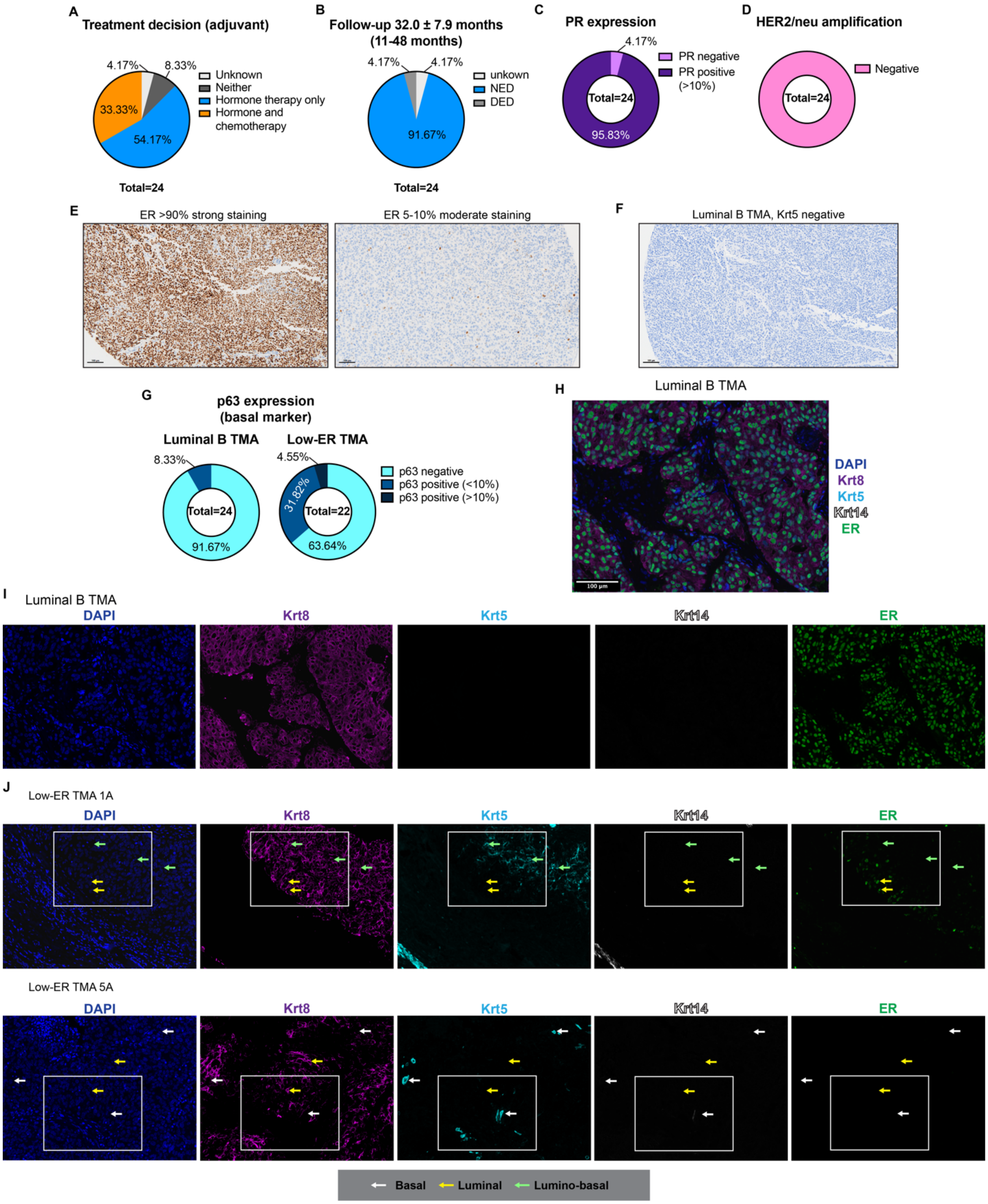

Supplementary figure 2: (A-D) Breakdown of treatment decisions **(A)**, follow-up details **(B)**, PR expression **(C)**, and Her2/neu amplification **(D)**, of the 24 luminal B cases used

in the TMA. **(E)** Representative IHC images showing the different levels and intensity of ER staining in the luminal B TMA cores. **(F)** Representative image of Krt5 IHC staining in luminal B TMAs. **(G)** p63 expression differences between luminal B and low-ER tumors. **(H-J)** Representative images of TSA staining showing homogeneous populations in luminal B TMAs and heterogeneous populations in low-ER TMAs. Samples were stained with Krt8 (purple), Krt5 (cyan), Krt14 (white), ER (green), and DAPI (blue). **(H)** Representative image of TSA staining showing homogeneous populations in luminal B TMAs. **(I)** Single channel images of luminal B TMA from Fig S2H. **(J)** Single channel images of low-ER TMAs from Fig 2H.

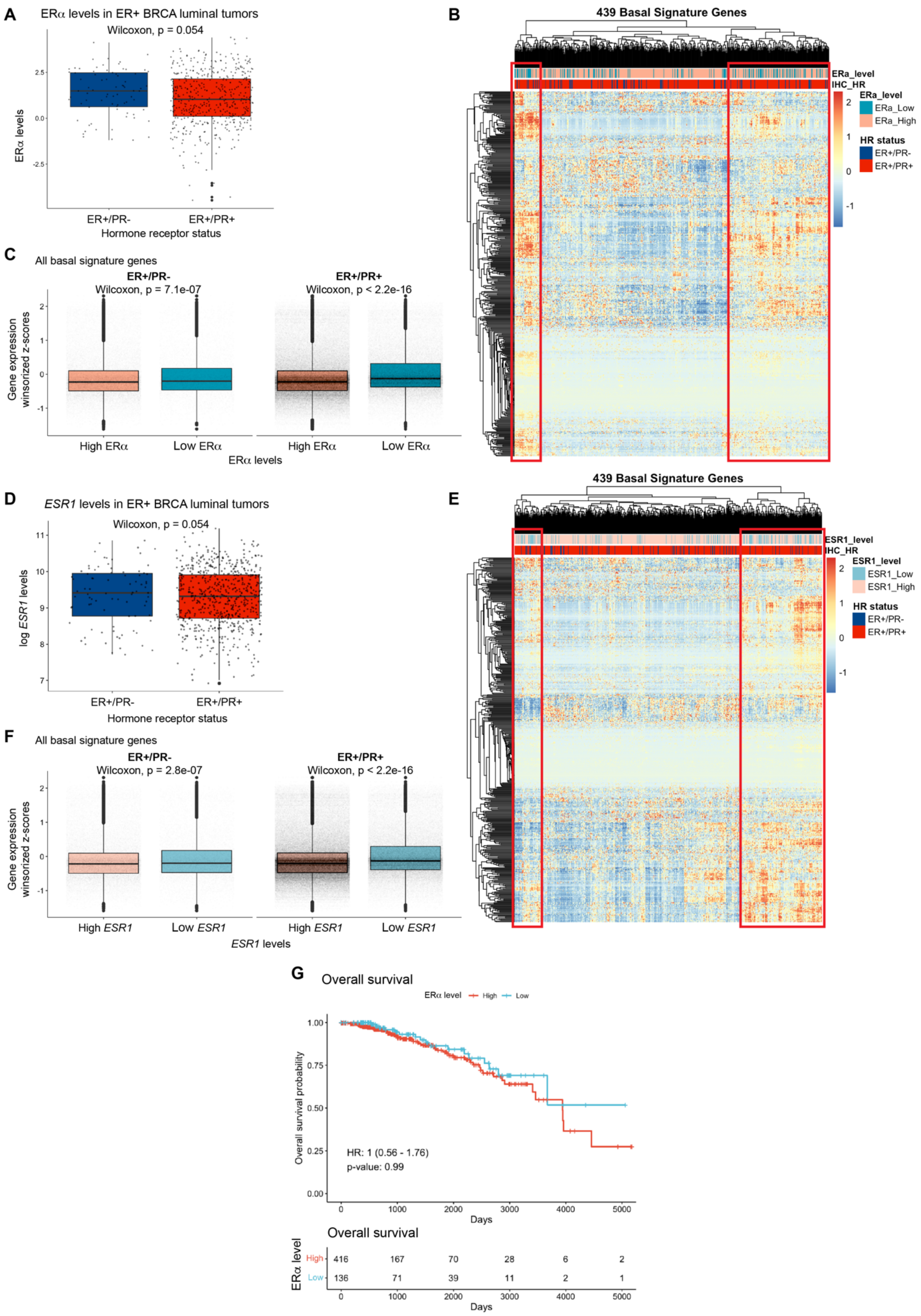
A
ERα levels in ER+ BRCA luminal tumors
Wilcoxon, p = 0.054
ERα levels
ER+/PR-
ER+/PR+
Hormone receptor status
B
439 Basal Signature Genes
ERa_level
IHC_HR
ERa_Low
ERa_High
HR status
C
All basal signature genes
Wilcoxon, p = 7.1e-07
Wilcoxon, p < 2.2e-16
Gene expression winsorized z-scores
High ERα
Low ERα
ERα levels
D
ESR1 levels in ER+ BRCA luminal tumors
log ESR1 levels
E
ESR1_level
ESR1_Low
ESR1_High
F
Wilcoxon, p = 2.8e-07
High ESR1
Low ESR1
ESR1 levels
G
Overall survival
ERα level
High
Low
Overall survival probability
HR: 1 (0.56 - 1.76)
p-value: 0.99
Days
416 167 70 28 6 2
136 71 39 11 2 1

Supplementary figure 3: (A) Boxplots showing distribution of ERα levels in the ER+/PR- and ER+/PR+ cases used in this analysis. **(B)** Heatmap with unsupervised clustering of the basal signature gene expression in ER positive tumors. **(C)** Boxplots showing the differences in distribution of basal signature gene expression stratified by ERα expression level and PR status. **(D)** Boxplots showing distribution of *ESR1* levels in the ER+/PR- and ER+/PR+ cases used in this analysis. **(E)** Heatmap with unsupervised clustering of the basal signature gene expression in ER positive tumors. **(F)** Boxplots showing the differences in distribution of basal signature gene expression stratified by *ESR1* expression level and PR status. **(G)** Kaplan-Meier plot of overall survival in ER positive breast cancer cases from TCGA stratified by ERα expression.

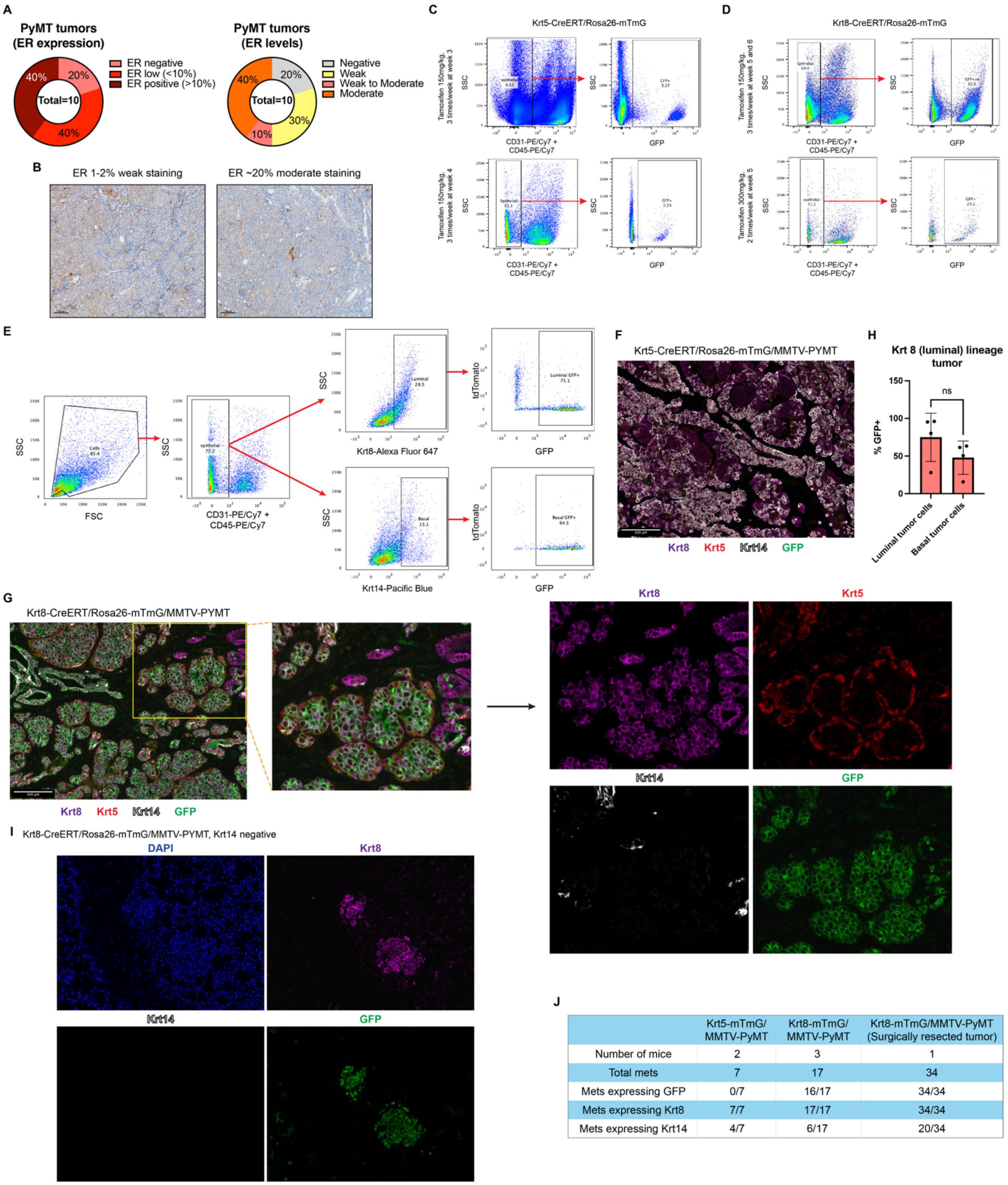

	Krt5-mTmG/ MMTV-PyMT	Krt8-mTmG/ MMTV-PyMT	Krt8-mTmG/MMTV-PyMT (Surgically resected tumor)
Number of mice	2	3	1
Total mets	7	17	34
Mets expressing GFP	0/7	16/17	34/34
Mets expressing Krt8	7/7	17/17	34/34
Mets expressing Krt14	4/7	6/17	20/34

Supplementary figure 4: (A) ER expression in MMTV-PyMT tumors. **(B)** Representative IHC images of ER expression in MMTV-PyMT tumors. **(C-D)** Optimization of tamoxifen induced GFP labelling in Krt5-CreERT/Rosa26-mTmG **(C)** and Krt8-CreERT/Rosa26-

mTmG **(D)** mouse mammary gland. **(E)** Flow cytometry gating strategy for identifying GFP expressing luminal and basal tumor cells. **(F-G)** Representative images of TSA staining of Krt5-CreERT/Rosa26-mTmG/MMTV-PyMT **(F)** and Krt8-CreERT/Rosa26-mTmG/MMTV-PyMT **(G)** tumors. Samples were stained with Krt8 (purple), Krt5 (red), Krt14 (white), and GFP (green). **(H)** Quantification of GFP expressing luminal and basal tumor cells from TSA stained images of Krt8-CreERT/Rosa26-mTmG/MMTV-PyMT tumors. **(I)** Single channel images of TSA staining of lung metastases from Krt8-CreERT/Rosa26-mTmG/MMTV-PyMT mice from Fig 4K showing no Krt14 expression. Samples were stained with Krt8 (purple), Krt14 (white), GFP (green), and DAPI (blue). **(J)** Counts of metastatic colonies from Krt5-CreERT/Rosa26-mTmG/MMTV-PyMT and Krt8-CreERT/Rosa26-mTmG/MMTV-PyMT mice expressing Krt8, Krt14, and GFP.

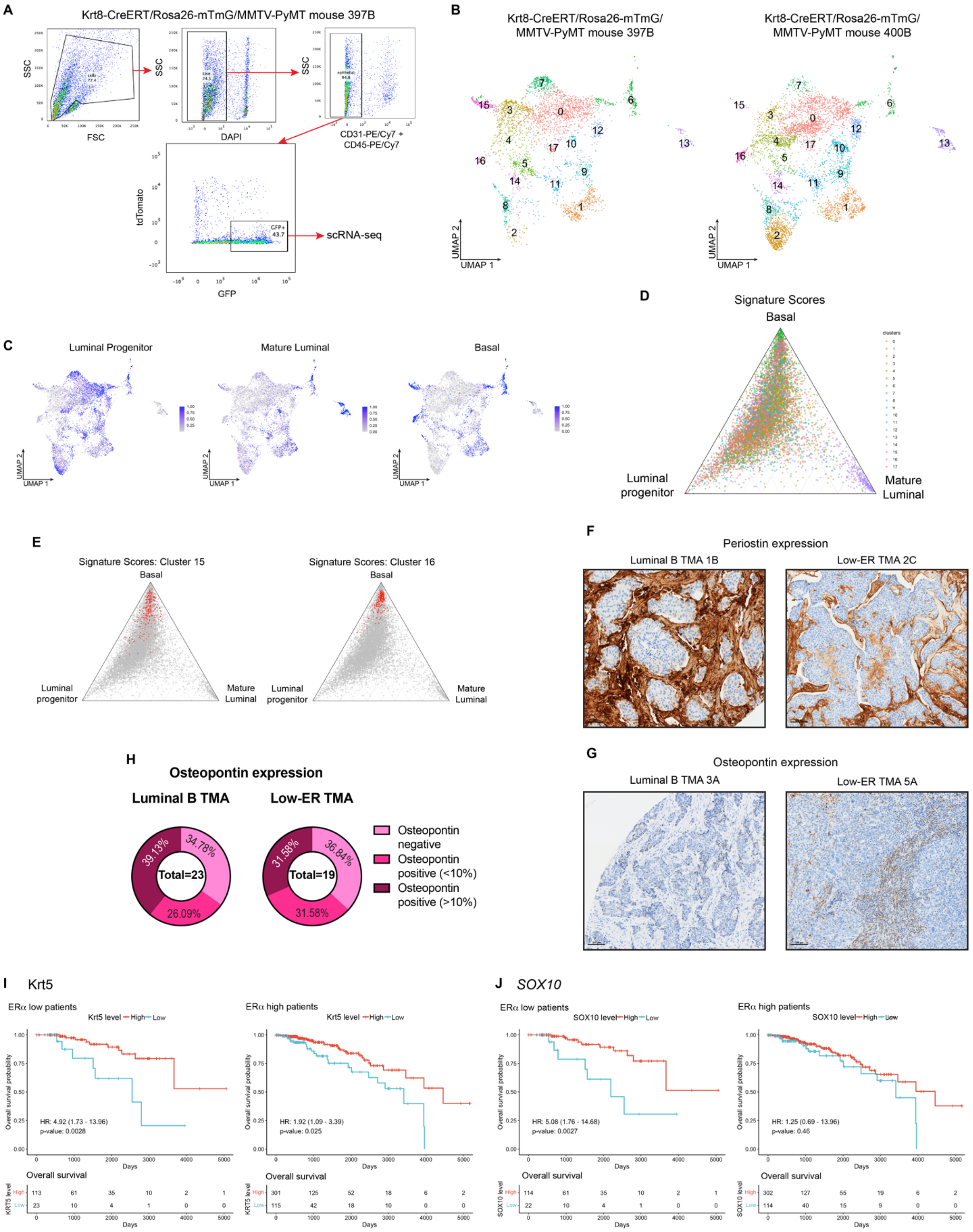
A
Krt8-CreERT/Rosa26-mTmG/MMTV-PyMT mouse 397B
SSC
FSC
DAPI
CD31-PE/Cy7 +
CD45-PE/Cy7
tdTomato
GFP
scRNA-seq
B
Krt8-CreERT/Rosa26-mTmG/
MMTV-PyMT mouse 397B
Krt8-CreERT/Rosa26-mTmG/
MMTV-PyMT mouse 400B
UMAP 1
UMAP 2
C
Luminal Progenitor
Mature Luminal
Basal
D
Signature Scores
Basal
Luminal
progenitor
Mature
Luminal
clusters
E
Signature Scores: Cluster 15
Signature Scores: Cluster 16
Basal
Luminal
progenitor
Mature
Luminal
F
Periostin expression
Luminal B TMA 1B
Low-ER TMA 2C
G
Osteopontin expression
Luminal B TMA 3A
Low-ER TMA 5A
H
Osteopontin expression
Luminal B TMA
Low-ER TMA
Total=23
Total=19
34.78%
26.09%
39.13%
36.84%
31.58%
31.58%
Osteopontin negative
Osteopontin positive (<10%)
Osteopontin positive (>10%)
I
Krt5
ERα low patients
ERα high patients
Krt5 level High Low
HR: 4.92 (1.73 - 13.96)
p-value: 0.0028
HR: 1.92 (1.09 - 3.39)
p-value: 0.025
J
SOX10
SOX10 level High Low
HR: 5.08 (1.76 - 14.68)
p-value: 0.0027
HR: 1.25 (0.69 - 13.96)
p-value: 0.46
Overall survival probability
Days
Overall survival
KRT5 level
SOX10 level

Supplementary figure 5: (A) Flow cytometry plots showing the gating strategy and the GFP positive cell population harvested using fluorescence assisted cells sorting (FACS). **(B)** UMAP of Krt8-CreERT/Rosa26-mTmG/MMTV-PyMT tumor cells sorted for GFP and split by sample of origin. Unsupervised clustering divided the cells into 18 different clusters. **(C)** Dimensionality reduction with UMAP of all tumor cells profiled with scRNA-seq. Cells are colored based on activity scores of luminal progenitor, mature luminal, and basal gene signatures. **(D)** Ternary plot showing the relative activity of luminal progenitor, mature luminal, and basal gene signatures across all tumor cells, colored by unsupervised cluster. **(E)** Ternary plots showing the distribution of relative activity of luminal progenitor, mature luminal, and basal gene signatures across all tumor cells profiled by scRNA-seq. In the left plot, cells assigned to cluster 15 are highlighted in red, while cells assigned to cluster 16 are highlighted in red in the right plot. **(F-G)** Representative images of periostin **(F)** and osteopontin **(G)** IHC staining in luminal B and low-ER TMAs. **(H)** Quantified osteopontin expression in luminal B and low-ER TMAs. **(I-J)** Kaplan-Meier plot of overall survival in ER positive breast cancer cases from TCGA stratified by Krt5 **(I)** and *SOX10* **(J)** expression in ERα low and ERα high cases.
